# Experimental and theoretical evaluation of the corrosion inhibition performance of two benzimidazole derivatives for low carbon steel in acidic solution

**DOI:** 10.1039/d5ra02749g

**Published:** 2025-07-14

**Authors:** Klodian Xhanari, Muhamed Farruku, Avni Berisha, Kledi Xhaxhiu, Jonida Canaj, Bujar Seiti, Efrosini Kokalari, Alketa Lame

**Affiliations:** a Faculty of Natural Sciences, University of Tirana Boulevard “Zogu I” Tirana 1001 Albania klodian.xhanari@unitir.edu.al; b Faculty of Natural and Mathematics Science, University of Pristina Pristina 10000 Kosovo

## Abstract

In this study, two benzimidazole derivatives, *i.e.* 2-(2-aminophenyl)-1*H*-benzimidazole (APhBI) and 2-(2-hydroxophenyl)-1*H*-benzimidazole (HPhBI) were tested as corrosion inhibitors for S235 steel in 1 M HCl solution, at 298–318 K. Weight loss, electrochemical impedance spectroscopy, and potentiodynamic polarization measurements were performed to evaluate the corrosion inhibition efficiency of these derivatives and the possible synergistic effect of five common intensifiers. The optimum corrosion inhibition concentration was found to be 3 mM for both derivatives, leading to corrosion inhibition efficiencies of 87.09% and 85.06% for APhBI and HPhBI, respectively. Electrochemical measurements revealed that after 24 h immersion both derivatives behaved mainly as cathodic-type inhibitors, following kinetically controlled processes. Attenuated total reflectance Fourier transform infrared spectroscopy and ultraviolet-visible spectroscopy measurements confirmed the adsorption of the compounds on the S235 steel samples, thus altering their morphology as observed by scanning electron microscopy measurements. Both physisorption and chemisorption are involved in the adsorption process, which obeys the Langmuir isotherm. Density Functional Theory (DFT), Monte Carlo (MC), and Molecular Dynamics (MD) simulations confirmed the formation of a stable protective layer on the Fe(110) surface, with inhibitors aligning to maximize interactions with Fe atoms. Mulliken charge analysis and electrostatic potential (ESP) mapping revealed that heteroatoms (N and O) serve as primary adsorption sites, facilitating strong molecular interactions with the metal surface.

## Abstract 2

Two benzimidazole derivatives, *i.e.* 2-(2-aminophenyl)-1*H*-benzimidazole (APhBI) and 2-(2-hydroxophenyl)-1*H*-benzimidazole (HPhBI) were found to successfully mitigate the corrosion of S235 steel in 1 M HCl solution, at 298–318 K.

## Graphical abstract

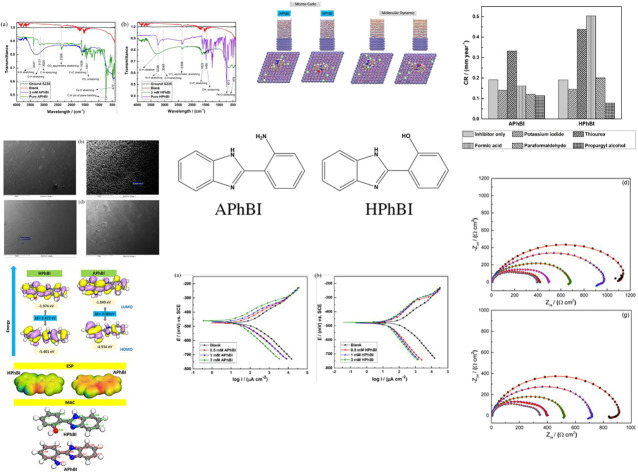

## 1. Introduction

Corrosion is a destructive phenomenon leading to material deterioration, thus raising serious safety, health, and environmental concerns, in addition to bringing significant economic costs.^1^ Although one of the most used materials, the highly desired physical and mechanical properties as well as the cost-effectiveness of carbon steel are to a degree overshadowed by its corrosion susceptibility in highly acidic environments which are often encountered in several industrial applications.^2,3^

A wide range of compounds and products, varying from inorganic to organic and to environmentally-friendly have been proven effective in mitigating the corrosion of carbon steel in acidic solutions.^3–8^ The N-heterocyclic compounds are among the most studied groups of organic compounds as corrosion inhibitors against the acid corrosion of carbon steel.^4,5,8–13^ In particular, benzimidazole derivatives owe their corrosion inhibition efficiency to their planar, fused bicyclic structure (comprised of benzene and imidazole rings) containing two nitrogen atoms.^9,10^ These derivatives interact with the metals through the π-electrons, and the lone electron pairs on the nitrogen atoms, in addition to those from other heteroatoms in the functional groups.^10^ As a result of this adsorption process a protective layer is formed on the surface of the carbon steel shielding it from the corrosion environment. Moreover, the solubility of benzimidazoles in polar solutions, including acidic environments, combined with their ease of functionalization, and relatively low toxicity, renders them very attractive in corrosion mitigation.^9,10,14^

The interaction between the inhibitors and metal surfaces is strongly related to length of the hydrocarbon chain, size of the atomic ring, and in particular to the presence of electron-donating groups.^15,16^ 2-Phenylbenzimidazole was found to be an effective corrosion inhibitor for high-strength X70 steel in 1 M HCl solution, at 308 K.^17^ The compound was adsorbed on the steel surface through chemisorption and physisorption mechanisms, affecting both the anodic and cathodic corrosion reactions. The effect of the substituent groups on the corrosion inhibition efficiency of mild steel in 1 M HCl solution was investigated by Zhang *et al.*.^18^ Starting from 2-mercaptobenzimidazole (MBI), two derivatives, *i.e.* 2-thiobenzylbenzimidazole (TBBI) and 1-butyl-2-thiobenzylbenzimidazole (BTBBI) were synthesized. The authors reported that the corrosion inhibition efficiency of these compounds follows the order BTBBI > TBBI > MBI. Zhu *et al.*^19^ reported a more pronounced decrease in the corrosion rate of the carbon steel samples in 1 M HCl solution upon addition of 0.05–5.00 mM of a newly synthesized surface active 2-aminobenzimidazole derivative (*i.e.* 2-(*n*-hexylamino)-4-(3-*N*,*N*-dimethylaminopropyl)amino-6-(2-aminobenzimidazol)-1,3,5-*s*-triazine), compared with the benzimidazole precursor. Moreover, the 2-aminobenzimidazole derivative showed improved corrosion inhibition performance at high temperatures. *o*-phenylenediamine is a common precursor used to obtain benzimidazole derivatives *via* the Weidenhagen method. The addition of 200 mg L^−1^ of the newly synthetized environmentally friendly compound 2-styryl-1*H*-benzo[*d*]imidazole decreased the corrosion current densities of carbon steel in 15% HCl solution from 1649 to 103 μA cm^−2^, reaching a maximum corrosion inhibition efficiency of 93.75%.^20^

The interest in studying the corrosion susceptibility of S235 steel (a low carbon, structural steel, with high Mn additions) in acidic solutions, is strongly related to its lower cost and high mechanical resistance which make this steel desirable in several applications, including in construction (equipment and different types of buildings), for gas, oil and water pipelines, and in marine industry.^21,22^ 2-(2-Aminophenyl)-1*H*-benzimidazole (APhBI) and 2-(2-hydroxophenyl)-1*H*-benzimidazole (HPhBI) are two derivatives that in addition to the benzimidazole core contain also two electron-donating groups, *i.e.* –NH_2_ and –OH, connected to the phenyl ring, which can further increase their adsorption ability on the steel surface.

Herein, the short- and moderate-term corrosion inhibition performance of APhBI and HPhBI for S235 steel samples in 1 M HCl solution was first evaluated in the temperature range 298–318 K, after 1 and 24 h immersion, respectively. The influence of the inhibitors' concentration, temperature and the addition of several intensifiers on the corrosion inhibition efficiency of these compounds was studied using the weight loss (WL), electrochemical impedance spectroscopy (EIS) and potentiodynamic curve polarization (PD) measurements. Next, Attenuated total reflectance Fourier transform infrared spectroscopy (ATR-FTIR) and scanning electron microscope (SEM) measurements were employed to first confirm the adsorption of the inhibitors, and then to understand their influence on the morphology of S235 steel samples. Finally, a combination of ultraviolet-visible spectroscopy (UV-Vis) measurements, thermodynamic studies and theoretical analysis (including MC and MD simulations as well as DFT calculations) was used to shed light on the corrosion inhibition mechanism of these compounds. Quantum chemical parameters were analysed to understand the electronic properties and adsorption behaviour of the inhibitors.

Understanding the corrosion inhibition mechanism of these compounds, especially the influence of the functional groups, can lead to the development of new corrosion inhibitors. The presence of the above-mentioned electron-donating groups allows easy functionalization of these compounds. Moreover, the combination of these compounds with common intensifiers provides important insight in the development of corrosion inhibition formulations which can be used to improve their corrosion inhibition performance in severe conditions (high temperature and/or long exposure times).

## 2. Materials and methods

### 2.1 Sample and solution preparation

S235 steel with the chemical composition: 0.072 wt% C, 0.570 wt% Mn, 0.180 wt% Si, 0.022 wt% P, 0.004 wt% S, 0.310 wt% Cu, 0.006 wt% N, 0.012 wt% Al, and the remainder Fe, was provided by Italinox (Tirana, Albania). Cylindrical- (50 mm height and 8 mm diameter) and disc-shaped (15 mm diameter) samples cut out of 16 mm S235 steel bars were first pretreated as previously described^23,24^ and then employed to perform the weight loss and electrochemical measurements, respectively.

**Fig. 1:**
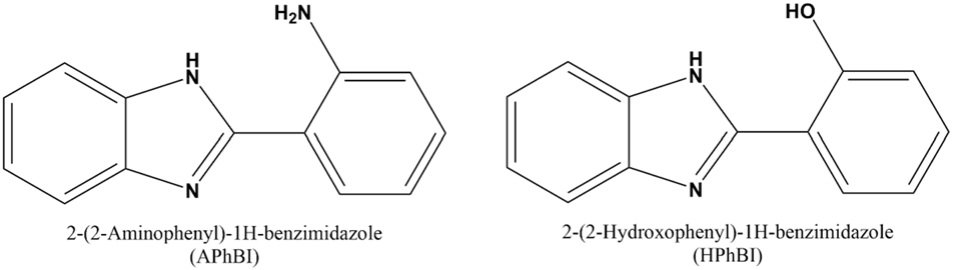
Structures of the benzimidazole derivatives tested in this study.

APhBI (97% purity) and HPhBI (98% purity), with structures presented in Fig. 1, were provided by Sigma-Aldrich (St. Louis, Missouri, USA), and BLDpharm (Shanghai, China), respectively. Bidistilled water and concentrated hydrochloric acid (HCl) provided by Carlo Erba (Milan, Italy) were used to prepare the 1 M HCl solution. Intensifiers, such as potassium iodide (KI), formic acid (FA), and paraformaldehyde (PFA) provided by VWR Chemicals (Lutterworth, UK), as well as thiourea (TU) and propargyl alcohol (PA) provided by Thermo Fisher Scientific (Massachusetts, USA) were added in a 3 : 1 inhibitor to intensifier ratio. These compounds enhance the efficiency or stability of a primary corrosion inhibitor by synergistically improving its adsorption, film-forming ability, or electrochemical performance in the corrosion environment.^25^ Their selection is based both on the type of inhibitors used and the corrosion environment.^25–27^ Acetone (for analysis-ISO-ACS) provided by Carlo Erba (Milan, Italy) was used to pretreat the S235 steel samples.

### 2.2 Weight loss (WL) measurements

Before immersion for 24 h in 1 M HCl solutions with and without additions of 0.5–3 mM of each inhibitor, the S235 steel samples were weighed and measured (to determine their surface, *S*). At the end of the immersion time the samples were treated as previously described^23,24^ (including removal of corrosion products, rinsing with bidistilled water and degreasing in acetone) and then weighed again. Eqn (1)–(4) were used to determine the corrosion rate (CR) of the S235 steel samples in (g m^−2^ h^−1^) and in (mm year^−1^), the corrosion inhibition efficiency (CIE_WL_ (%)), and surface coverage (*θ*) of the inhibitors, respectively.1
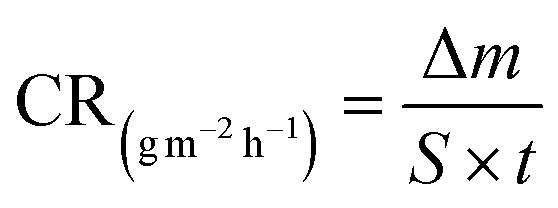
2
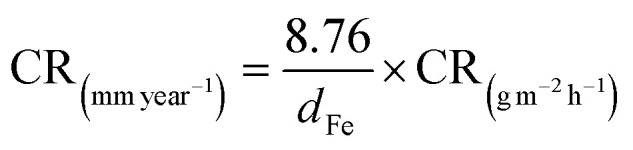
3
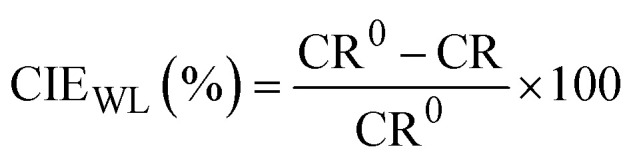
4
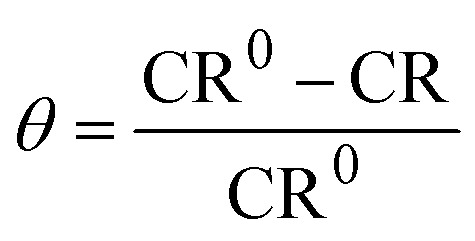
Herein, Δ*m* is the average weight loss of the samples before and after immersion in the corrosion environment and *t* is the immersion time. At least three replicate measurements have been performed for each system. The corrosion rates of the steel samples with and without addition of the inhibitors are denoted as CR and CR^0^, respectively. The conversion between the two units of CR is done using the density of Fe as *d*_Fe_ = 7.86 g cm^−3^, and the conversion coefficient as 8.76.

The influence of inhibitors' concentration on the corrosion susceptibility of the S235 steel samples was studied at 298 K. Meanwhile, the influence of temperature on the corrosion susceptibility of the S235 steel samples was studied in the 298–318 K range, only in 1 M HCl solutions containing the optimum corrosion inhibition concentration (OCIC) of each inhibitor. The obtained results are used to plot the best fitting adsorption isotherm and to obtain the Arrhenius and transition state plots from which the activation parameters are determined.

### 2.3 Electrochemical measurements

A Palmsens 4 potentiostat/galvanostat and a three-electrode cell from PalmSens (Houten, Netherlands) consisting of S235 steel (working electrode), Pt (counter electrode) and saturated calomel electrode, SCE (reference electrode). All the electrochemical measurements were performed at 298 K.

First, the S235 steel samples were immersed in the 1 M HCl solutions with and without additions of each inhibitor and intensifiers to stabilize for 1 h and the open-circuit potential (*E*_OC_) was recorded. Then, for short-term measurements, the potentiodynamic polarization (PD) curves were obtained. In the case of moderate-term measurements, the electrochemical impedance spectroscopy (EIS) spectra of the S235 steel samples were recorded next after 1, 5, 10, 15, 20, and 24 h immersion in the 1 M HCl solutions containing the OCIC and 1 mM of the intensifiers. A 10 points per decade and 10 mV amplitude excitation signal was used to obtain the EIS response in the 100 kHz to 10 mHz frequency range. Several equivalent electrical circuits (EEC) and the PSTrace 5.11 software from PalmSens (Houten, Netherlands) were used to fit the obtained EIS spectra. *E*_OC_ was measured in between the EIS measurements and for 10 min prior to the moderate-term PD curve measurements. Finally, a 0.5 mV s^−1^ potential scan rate was employed to obtain the PD curves of the S235 steel samples in the *E*_OC_ ± 120 mV potential range. The corrosion current density values with and without additions of the inhibitors and intensifiers (*i.e. i*_corr_ and *i*^0^_corr_, respectively), obtained from the PD curve measurements, were used to calculate the CR (mm year^−1^) of the S235 steel samples and the CIE_PD_ (%) of the inhibitors.5
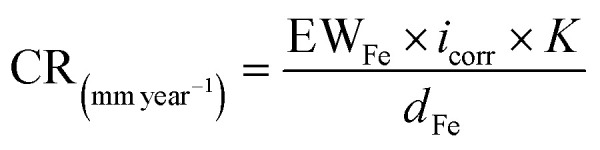
6
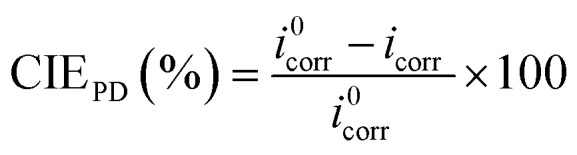
where EW_Fe_ ≈ 27.92 is the equivalent weight in the case of Fe^2+^ and *K* = 0.00327 is the proportionality constant.

### 2.4 UV-Vis measurements

A PhotoLab 7600 UV-VIS series from Xylem Analytics (California, USA) was used to perform the UV-Vis measurements. The full absorption spectra (*i.e.* in the 190–1100 nm range) of the OCIC solution of each inhibitor was obtained before and after 24 h immersion of the S235 steel samples, at 298 K. Prior to the measurements, each solution was diluted (1 : 100) to ensure reasonable absorbance values.

### 2.5 Surface analysis

ATR-FTIR and SEM measurements were performed on the S235 steel samples before and after immersion for 72 h in 1 M HCl solutions with and without additions of the inhibitors. Alpha II spectrometer from Bruker (Massachusetts, USA) was used to obtain the ATR-FTIR spectra in the 400–4000 cm^−1^ range of the S235 steel samples and the pure inhibitors, for comparison. SEM micrographs of the samples were obtained with a SEM model EVO MA10 from Zeiss (Jena, Germany).

### 2.6 Theoretical analysis

#### 2.6.1 DTF calculations

The DMol^3^ software was employed to perform Density Functional Theory (DFT) calculations.^28–30^ The M06-L functional was selected to account for long-range adjusted exchange–correlation effects^31^ to accurately describe non-covalent interactions. To ensure a well-balanced electronic structure representation, the DNP basis set^32,33^ was applied to all atoms.^28^ The computations were conducted in a solvated environment using the COSMO model, with water as the solvent.^34–36^ This approach effectively captures solvation effects by simulating the solvent as a continuous polarizable medium. For optimization, stringent convergence criteria were imposed to ensure precise results. Default fine settings were used for numerical integration grids and other computational parameters.

#### 2.6.2 MC and MD simulations

This study explored how the inhibitor interacts with an iron (Fe) substrate in both its neutral and protonated states, employing Monte Carlo (MC) and Molecular Dynamics (MD) simulations. The Forcite module within the Materials Studio (MatS) package was used to carry out the simulations.^37,38^ The system was modeled within a simulation box measuring 2.4823 nm × 2.4823 nm × 5.32416 nm, which included a 3.5 nm vacuum region, and periodic boundary conditions were applied.^39–42^ The Fe(110) surface,^43,44^ known for its high stability among common Fe crystallographic surfaces, was chosen as the representative model. To simulate the system, a slab model consisting of 10 atomic layers and a total of 1000 Fe atoms was constructed.^45^ The methodology for MD simulations followed standard approaches previously described in the literature.^24^ The simulated environment included one inhibitor molecule, 700 water molecules, five hydronium ions, and five chloride ions. The COMPASS III force field^46^ was applied to ensure accurate representation of interatomic interactions.^39,40,47,48^ The simulations were conducted within the NVT ensemble at a temperature of 295 K, using a time step of 1 femtosecond (fs) and a total simulation time of 1500 picoseconds.^37,38,49,50^

## 3. Results and discussions

### 3.1 Weight loss measurements

Table 1 presents the variation of the CR of the S235 steel samples after 24 h immersion in 1 M HCl solutions with and without APhBI and HPhBI additions, obtained from the WL measurement at 298 K.

**Table 1:** Influence of the concentration of APhBI and HPhBI on the corrosion rate of S235 steel samples immersed for 24 h in 1 M HCl solution, evaluated from the WL measurements performed at 298 K (including the standard deviation of at least three replicate measurements)

Solutions		CR (g m^−2^ h^−1^)	CR (mm year^−1^)	*θ*	CIE_WL_ (%)
Blank	—	0.996 ± 0.029	1.109 ± 0.032	—	—
APhBI	0.5 mM	0.335 ± 0.024	0.373 ± 0.027	0.664	66.4
	1 mM	0.262 ± 0.023	0.292 ± 0.026	0.737	73.7
	3 mM	0.146 ± 0.020	0.162 ± 0.022	0.854	85.4
HPhBI	0.5 mM	0.269 ± 0.028	0.300 ± 0.031	0.730	73.0
	1 mM	0.242 ± 0.016	0.269 ± 0.018	0.757	75.7
	3 mM	0.167 ± 0.019	0.186 ± 0.021	0.832	83.2

For both inhibitors, the CR decreased significantly with the increase in the inhibitors' concentration, from 0.996 g m^−2^ h^−1^ to 0.146 and 0.167 g m^−2^ h^−1^, for APhBI and HPhBI, respectively. This decrease is due to the increase in the surface coverage (*θ*) of the S235 steel samples by the molecules of the inhibitors. The layer formed on the surface of the samples isolates them from the corrosion environment. The increase in the concentration of the inhibitors resulted also in an increase of the CIE (%), reaching up to 85.4 and 83.2%, for the OCIC of APhBI and HPhBI, respectively. No significant change in the CR of the S235 steel samples was observed upon further addition of both inhibitors (*i.e.* 5 mM of each inhibitor was added, results not shown), indicating that 3 mM is the OCIC.

WL measurements performed in the temperature range 298–318 K showed that the corrosion inhibition performance of both inhibitors (*i.e.* using their OCIC) is significantly impacted by temperature (Table 2). At higher temperatures the surface coverage of the inhibitors on the S235 steel samples decreases. This can be attributed to the desorption of the inhibitors' molecules and to their reduced ability to adsorb due to the increase in the coarseness of the steel surface with increasing temperature.^51–54^ The latter can also cause rearrangement of the inhibitors' molecules, exposing this way the steel samples to the corrosion environment, leading to higher CR values as well as lower CIE (%) values for both inhibitors.

**Table 2:** Influence of temperature on the CR of S235 steel samples immersed for 24 h in 1 M HCl solution containing 3 mM APhBI and HPhBI, evaluated from the WL measurements performed at the 298–318 K range (including the standard deviation of at least three replicate measurements)

Solutions	298 K		308 K		318 K	
	CR (g m^−2^ h^−1^)	CIE_WL_ (%)	CR (g m^−2^ h^−1^)	CIE_WL_ (%)	CR (g m^−2^ h^−1^)	CIE_WL_ (%)
Blank	0.996 ± 0.029	—	3.596 ± 0.095	—	16.105 ± 0.216	—
3 mM APhBI	0.146 ± 0.020	85.4	0.738 ± 0.031	79.5	6.031 ± 0.136	62.6
3 mM HPhBI	0.167 ± 0.019	83.2	0.868 ± 0.033	75.9	6.734 ± 0.152	58.2

### 3.2 Electrochemical measurements

EIS and PD curve measurements were used to evaluate the corrosion inhibition performance of APhBI and HPhBI for S235 steel in 1 M HCl solution at 298 K. Short-term measurements (*i.e.* 1 h immersion) have been first performed to confirm the optimum concentration of the inhibitors. Next, several intensifiers were added to the OCIC of both inhibitors, and the short-term performance of the inhibitors was tested. Finally, the influence of immersion time (up to 24 h immersion) on the corrosion inhibition performance of the optimum concentration of each inhibitor was studied.

#### 3.2.1 Short-term measurements

**Fig. 2:**
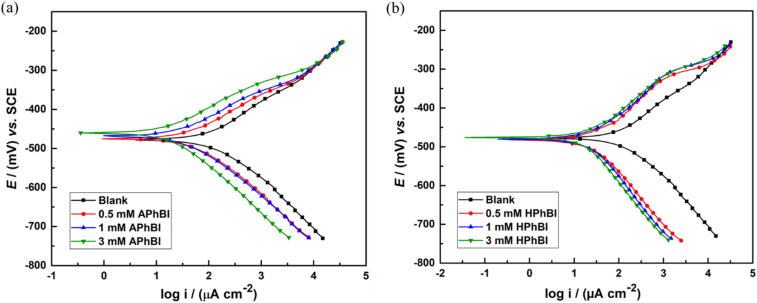
PD curves of the S235 steel samples immersed for 1 h in 1 M HCl solutions with and without addition of 0.5–3 mM of (a) APhBI and (b) HPhBI, respectively.

The PD curves of the S235 steel samples after 1 h immersion in 1 M HCl solutions with and without additions of each inhibitor are presented in Fig. 2. The addition of 0.5, 1, and 3 mM of both APhBI (Fig. 2a) and HPhBI (Fig. 2b) shifted both the cathodic and anodic branches of the PD curves of the S235 steel samples immersed for 1 h in 1 M HCl solutions at 298 K to lower corrosion current density values (*i*_corr_) compared with the uninhibited solutions. As a result, both inhibitors behave as mixed-type inhibitors. Nevertheless, Fig. 2 shows that the addition of both inhibitors resulted in a more pronounced influence mostly on the cathodic corrosion reaction. This is in accordance with the corrosion inhibition mechanism of 2-phenylbenzimidazole on X70 steel, previously reported by Ran *et al.*,^17^ as well as with that proposed by Zhang *et al.*^18^ for the three benzimidazole derivatives (*i.e.* MBI, TBBI and BTBBI) on mild steel, both immersed in 1 M HCl solution.

When increasing the concentration of APhBI the corrosion potential (*E*_corr_) of the S235 samples moved to less negative values (Table 3). Meanwhile, the addition of 0.5 mM HPhBI shifted the *E*_corr_ to more negative values compared with that in the uninhibited solution. However, upon further addition of HPhBI, the *E*_corr_ did not significantly change. For both inhibitors, |Δ*E*_corr_| < 85 mV, confirming that APhBI and HPhBI behave as mixed-type inhibitors.^55,56^ Increasing the concentration of both inhibitors from 0.5 to 3 mM resulted in decreased *i*_corr_ (*i.e.* from 111.37 μA cm^−2^ to 14.38 and 16.64 μA cm^−2^ for APhBI and HPhBI, respectively). Consequently, the CR of the S235 steel samples decreased also from 1.159 g m^−2^ h^−1^ to 0.1497 and 0.173 g m^−2^ h^−1^, for APhBI and HPhBI, respectively. The decrease of CR at the lowest concentration was more pronounced for HPhBI compared with APhBI. The efficiency of both inhibitors mitigating the corrosion of S235 steel samples in 1 M HCl is also seen by the increase in the *R*_p_ values (Table 3).

**Table 3:** Corrosion parameters obtained from the PD curves of S235 steel samples immersed for 1 h in 1 M HCl solutions with and without addition of 0.5–3 M APhBI and HPhBI (including the standard deviation of at least three measurements)

Solutions		*E*_corr_ (mV)	*b*_c_ (mV dec^−1^)	*b*_a_ (mV dec^−1^)	*i*_corr_ (μA cm^−2^)	CR (g m^−2^ h^−1^)	CR (mm year^−1^)	CIE_PD_ (%)
Blank	—	−475.4 ± 5.0	−106.3 ± 3.5	101.4 ± 5.2	111.4 ± 3.7	1.159 ± 0.039	1.290 ± 0.043	—
APhBI	0.5 mM	−470.5 ± 4.6	−104.4 ± 5.4	84.6 ± 4.9	43.3 ± 3.2	0.450 ± 0.033	0.501 ± 0.037	61.2
	1 mM	−463.7 ± 6.2	−104.1 ± 5.9	82.7 ± 3.8	33.1 ± 4.9	0.344 ± 0.051	0.383 ± 0.057	70.3
	3 mM	−458.6 ± 5.8	−100.2 ± 3.6	81.0 ± 6.1	14.4 ± 3.5	0.150 ± 0.036	0.166 ± 0.040	87.1
HPhBI	0.5 mM	−490.2 ± 6.3	−103.9 ± 5.5	99.4 ± 5.4	21.8 ± 3.4	0.227 ± 0.035	0.253 ± 0.039	80.4
	1 mM	−493.8 ± 5.4	−100.6 ± 3.9	100.2 ± 3.7	19.0 ± 2.1	0.198 ± 0.022	0.220 ± 0.024	82.9
	3 mM	−493.7 ± 4.6	−99.7 ± 4.2	101.7 ± 5.6	16.6 ± 2.4	0.173 ± 0.025	0.193 ± 0.028	85.1

Table 3 showed that the CIE (%) of both inhibitors increased with increasing their concentration reaching their maximum values upon addition of 3 mM inhibitor (*i.e.* 87.1 and 85.1% for APhBI and HPhBI, respectively). No significant change in the CR rate of the S235 steel samples was observed for higher concentrations of the inhibitors (*i.e.* 5 mM of each inhibitor was tested, results not shown) confirming that 3 mM is the OCIC for both inhibitors.

Five of the most common intensifiers,^26^ including potassium iodide (KI), thiourea (TU), formic acid (FA), paraformaldehyde (PFA), and propargyl alcohol (PA) were added to the 1 M HCl solutions containing the OCIC of each inhibitor in a 3 : 1 inhibitor to intensifier ratio. The respective CRs the S235 steel samples were determined from the short-time PD curve measurements.

**Fig. 3:**
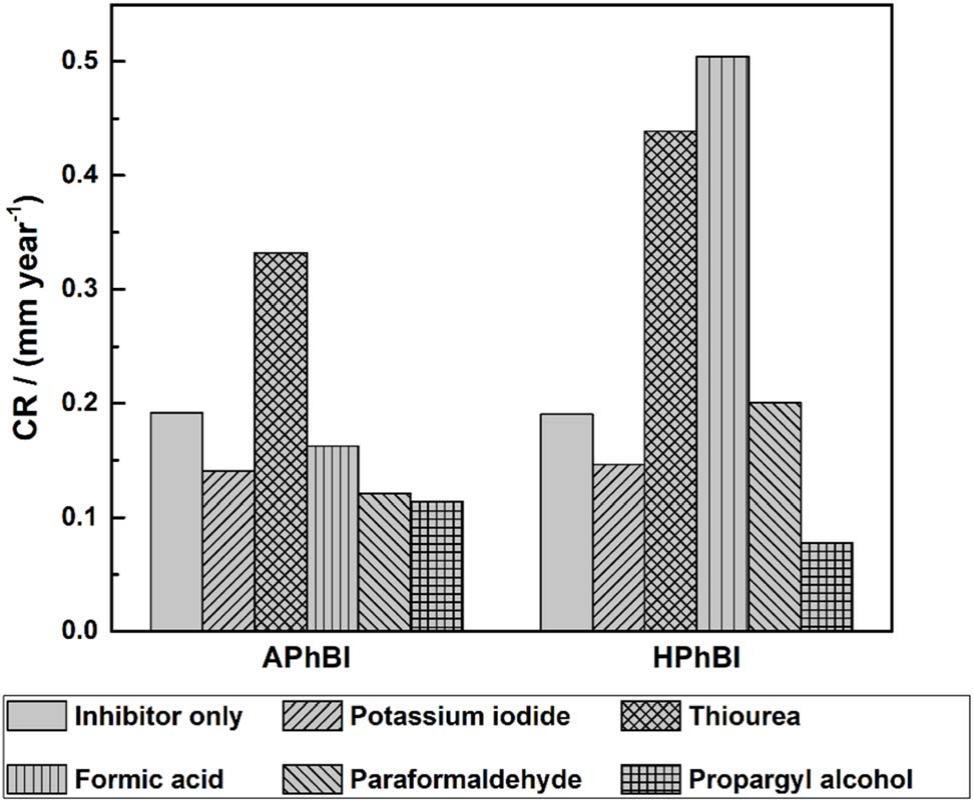
Variation of the CR of the S235 steel samples immersed for 1 h in 1 M HCl solutions containing 3 mM of each inhibitor, in addition to 1 mM of KI, TU, FA, PFA, and PA.

Fig. 3 presents the influence of these compounds on the corrosion inhibition efficiency of each inhibitor. For APhBI, with the exception of TU, the addition of 1 mM of all the intensifiers further decreased the CR of the S235 steel samples. Meanwhile, for HPhBI only KI and PA were found effective in improving its corrosion inhibition performance. PA was found to be the most effective intensifier for both inhibitors.

#### 3.2.2 Influence of immersion time on the corrosion inhibition performance

**Fig. 4:**
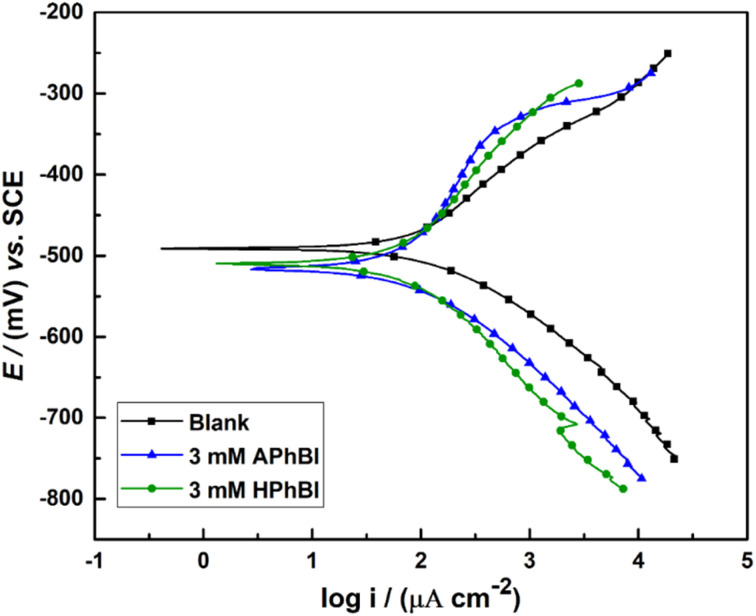
PD curves of the S235 steel samples immersed for 24 h in 1 M HCl solutions with and without the addition of the OCIC of APhBI and HPhBI.

Fig. 4 presents the PD curves of the S235 steel samples immersed for 24 h in 1 M HCl solutions containing 3 mM of APhBI and HPhBI. Both inhibitors provided moderate corrosion protection of the S235 steel samples after 24 h immersion in 1 M HCl solution.

No significant change in the CIE (%) of both inhibitors was observed. Moreover, both APhBI and HPhBI behaved as cathodic type inhibitors.

**Fig. 5:**
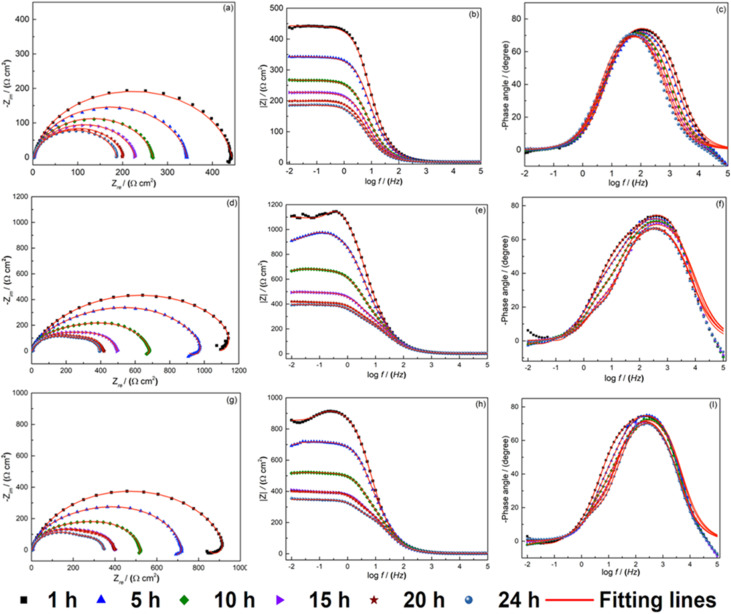
The EIS response (measured and fitted) of the S235 steel samples after 1, 5, 10, 15, 20, and 24 h immersion in 1 M HCl solution, at 298 K, containing: (a–c) no addition of the inhibitors, (d–f) 3 mM HPhBI, and (g–i) 3 mM APhBI.

The corrosion susceptibility of the S235 steel samples in 1 M HCl solution with and with addition of the OCIC of HPhBI and APhBI was evaluated with EIS after 1, 5, 10, 15, 20, and 24 h immersion at 298 K. The Nyquist plots are displayed in Fig. 5a, d and g. In the case of the uninhibited solution only one capacitive loop is observed, indicating that the charge transfer process predominates at the metal/solution interface.^23^

The slightly depressed capacitive loops are attributed to the inhomogeneity and surface roughness of the S235 steel samples.^57^

For solutions containing inhibitors at optimum concentration, the presence of inductance (*L*) persists for the first 15 h of immersion. This is due to the formation of corrosion products that are unstable on the metal solution interface.^23^ The Bode plots of the samples obtained after 20 h immersion in inhibited solutions (Fig. 5d,g) display the development of two semicircles. This indicate the formation of a film layer on the surface of metal surface with the inhibitor molecules and the presence of charge transfer resistance on the metal solution interface.^58^ The Bode plot spectra (Fig. 5b, e and h) for samples immersed in uninhibited and inhibited solutions reveal no significant changes in shape over time, indicating a consistent inhibition mechanism.^59^ Furthermore, Bode plots decrease with time for blank and inhibitors solution indicating an increase of corrosion rate with time. The phase angle spectra consistently display a single peak, for blank solution and two peaks (a strong one at high frequencies and a weak one at low frequencies) for inhibited solutions confirming the finding on Nyquist spectra.

**Fig. 6:**
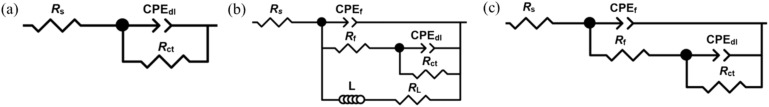
EEC used to fit the EIS response of the S235 steel samples in 1 M HCl solutions containing: (a) no addition of the inhibitors, (b) the OCIC of the inhibitors recorded after 1–10 h immersion, (c) the OCIC of the inhibitors recorded after 15–24 h immersion.

The experimental data of EIS measurements were fitted based on the lowest goodness of the fitting procedure (*χ*^2^), three electrochemical equivalent circuits (EECs) (Fig. 6) were employed to model the EIS data for the blank (Fig. 5a–c), HPhBI and APhBI solutions (Fig. 5d–f as well as Fig. 5g–i, respectively). All circuits contain solution resistance (*R*_s_), charge transfer resistance (*R*_ct_), and a constant phase element (CPE). The EECs for the inhibited solutions additionally include a film resistance (*R*_f_), inductance (*L*), resistance of inductance (*R*_L_), and a film-associated constant phase element (CPE_f_), corresponding to the protective layer formed by HPhBI and APhBI molecules. The impedance of a CPE is defined as:^60^7
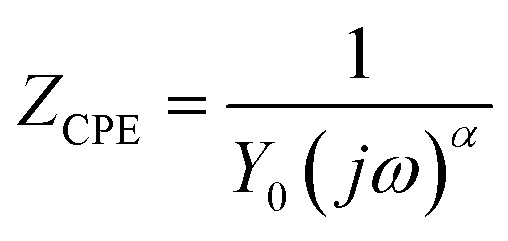
where *Y*_0_ is the proportionality factor, *ω* (angular frequency) is defined as 2π*f*, and *α* quantifies the deviation from ideal capacitive behaviour (*α* = 1 for a pure capacitor, *α* = 0 for a resistor, and *α* = −1 for an inductor).

The double-layer capacitance (*C*_dl_) and film capacitance (*C*_f_) are derived from the following equations:^48^8
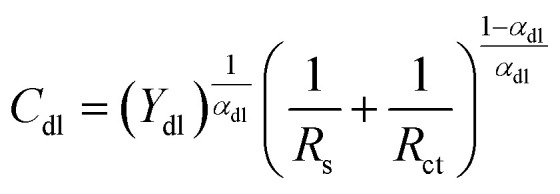
9
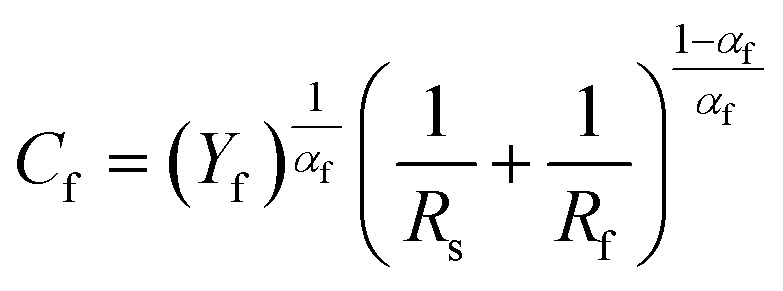


Table 4 summarizes the data found from the fitted electrochemical parameters with the respective circuits. For the blank solutions, *R*_ct_ decreased over time, reflecting an increased corrosion rate. In contrast, the addition of 3 mM HPhBI and APhBI led to a higher overall resistance (*R*_t_ = *R*_f_ + *R*_ct_), revealing the inhibition of the corrosion reaction.

**Table 4:** Parameters obtained from fitting the EIS response of the S235 steel samples immersed for 1, 5, 10, 15, 20, and 24 h immersion in 1 M HCl solutions with and without additions of 3 mM of APhBI and HPhBI, using the EECs presented in Fig. 6

Time (h)	Solutions	*R*_s_ (Ω cm^2^)	*C*_f_ (μF cm^−2^)	*α*_1_	*R*_f_ (Ω cm^2^)	*C*_dl_ (μF cm^−2^)	*α*_2_	*R*_ct_ (Ω cm^2^)	*R*_t_ (Ω cm^2^)	*L* (kH cm^2^)	*R*_L_ (Ω cm^2^)	*χ* ^2^ (×10^−3^)
1	Blank	2.11	—	—	—	24.09	0.91	440.99	440.99	—	—	3.0
	3 mM APhBI	2.21	10.78	0.92	373.98	0.74	0.72	564.95	938.93	18.290	9339	2.2
	3 mM HPhBI	2.02	8.71	0.94	192.26	0.22	0.65	1125.96	1318.22	5.027	6902	1.6
5	Blank	2.25	—	—	—	28.05	0.90	341.1	341.1	—	—	2.8
	3 mM APhBI	2.09	10.17	0.91	497.05	49.66	0.92	220.69	717.74	169.344	7941	3.4
	3 mM HPhBI	1.81	7.09	0.88	428.01	5.66	0.76	573.58	1001.59	47.050	9773	3.6
10	Blank	2.09	—	—	—	42.53	0.90	265.28	265.28	—	—	4.8
	3 mM APhBI	2.04	10.11	0.89	365.11	119.79	0.94	154.54	519.65	192.364	2818	2.9
	3 mM HPhBI	1.66	6.63	0.86	398.39	38.62	0.85	286.04	684.43	200.242	7199	5.2
15	Blank	2.08	—	—	—	51.76	0.89	225.81	225.81	—	—	2.1
	3 mM APhBI	2.35	12.84	0.90	306.47	455.48	1.0	88.26	394.73	—	—	3.6
	3 mM HPhBI	1.64	6.27	0.85	337.05	137.37	0.91	157.96	495.01	—	—	4.2
20	Blank	2.07	—	—	—	63.57	0.89	198.82	198.82	—	—	4.5
	3 mM APhBI	2.19	12.81	0.90	300.49	473.88	0.97	95.27	395.76	—	—	1.2
	3 mM HPhBI	1.96	6.55	0.84	319.73	530.36	1.0	93.59	413.32	—	—	3.8
24	Blank	1.92	—	—	—	83.21	0.90	184.85	184.85	—	—	5.2
	3 mM APhBI	2.29	12.19	0.90	262.42	603.82	0.98	83.27	345.69	—	—	4.2
	3 mM HPhBI	1.98	6.77	0.84	294.77	544.15	0.98	99.41	394.18	—	—	6.1

The *R*_t_ values for the inhibited samples decreased but remained higher compared to the blank solution, indicating the development of a stable passivation layer on the steel surface. Table 4 shows an increase of *C*_dl_ values when increasing the immersion time up to 10 hours. This decrease indicates desorption of the inhibitor molecules. After 15 hours of immersion up a more stable value of *C*_dl_ is observed. This is supported by the disappearance of the inductive effect (*L*, *R*_L_), revealing the formation of a compact and stable film on the surface of the samples in the presence of inhibitor molecules. The adsorption of HPhBI and APhBI molecules at the metal/solution interface delayed the charge transfer process, thereby impeding corrosion.

### 3.3 Thermodynamic studies

As indicated by the findings of the weight loss and electrochemical methods, the surface layer responsible for isolating the steel samples from the corrosion environment is formed through the adsorption of the tested inhibitors. Therefore, to better understand the mechanism of action of the inhibitors, including the nature and mechanism of the adsorption process, plotting of different types of adsorption isotherms and calculation of thermodynamic activation parameters was performed.

#### 3.3.1 Adsorption isotherms

Several types of adsorption isotherms, including Langmuir, El-Awady, Flory–Hoggins, Freundlich, Temkin, and Frumkin were plotted using the WL measurements presented in Table 1. The coefficient of determination, *R*^2^ was used to confirm that Langmuir isotherm was found to be the best fit for both inhibitors. This was also confirmed by the fact that the slopes of the obtained isotherms are close to one.^61^ As was previously reported for other benzimidazole derivatives^17,20^ these types of compounds block the active sites on the steel surface through monolayer formation.

**Fig. 7:**
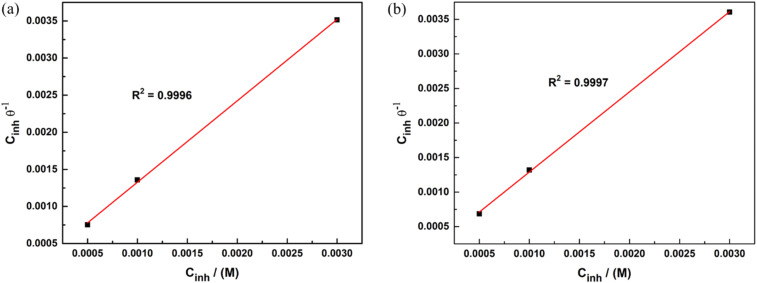
Langmuir adsorption isotherms for (a) APhBI and (b) HPhBI on S235 steel after 24 h immersion in 1 M HCl solution at 298 K.

Fig. 7 presents the Langmuir adsorption isotherms of APhBI and HPhBI, plotted according to eqn (10):10
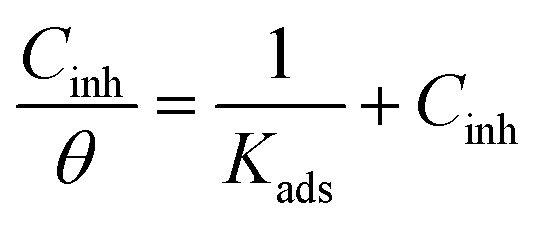
where *θ* is the surface coverage, *C*_inh_ is the concentration of the inhibitor (*i.e.* 0.5–3 mM), and *K*_ads_ is the adsorption equilibrium constant. *K*_ads_ is determined from the intercept of the Langmuir adsorption isotherm (Fig. 7). The higher this constant the stronger the inhibitor adsorbs on the steel surface.

The standard adsorption free energy (Δ*G*^0^_ads_) is calculated from eqn (11) using the obtained *K*_ads_ values for each inhibitor, the universal gas constant (*R* = 8.314 J mol^−1^ K^−1^), the absolute temperature (*T*) and the concentration of water at 298 K (55.5 mol L^−1^).

11 Δ*G*^0^_ads_ = −*RT* ln(55.5 × *K*_ads_)

The calculated Δ*G*^0^_ads_ for APhBI and HPhBI were −30.71 kJ mol^−1^ and –32.13 kJ mol^−1^, respectively, indicating in both cases spontaneous adsorption. Since the obtained Δ*G*^0^_ads_ values of each inhibitor are 20 kJ mol^−1^ < |Δ*G*^0^_ads_| < 40 kJ mol^−1^, both chemisorption and physisorption are possible and APhBI and HPhBI are considered mixed-type inhibitors,^62^ in accordance with what was previously reported for other benzimidazole derivatives.^17^

#### 3.3.2 Thermodynamic activation parameters

**Fig. 8:**
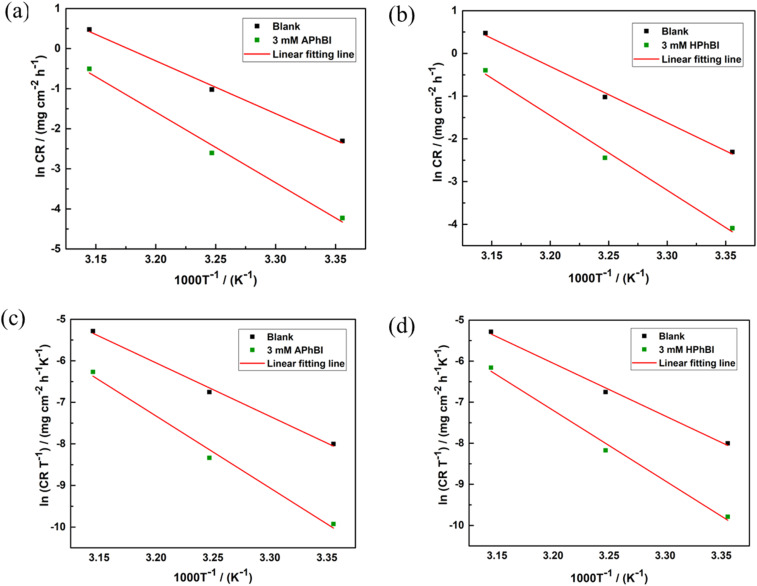
(a and b) Arrhenius plots for APhBI and HPhBI, respectively; (c and d) transition state plots for APhBI and HPhBI, respectively.

Determination of the activation energy (*E*_a_), entropy (Δ*S*_a_), and enthalpy (Δ*H*_a_) sheds further light into the corrosion inhibition performance of the tested inhibitors. The WL measurements performed in the temperature range 298–318 K for the OCIC of each inhibitor (Table 2) were used to plot the Arrhenius (Fig. 8a and b) and transition state (Fig. 8c and d) plots, according to eqn (12) and (13):12
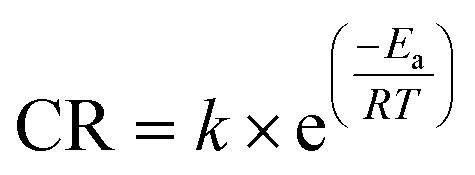
13
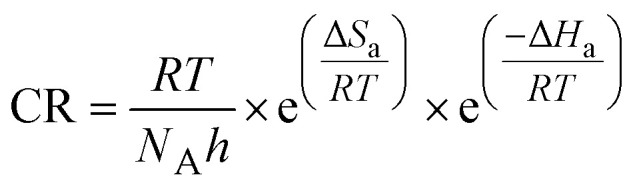
where *k* is the frequency factor, *h* = 6.62607015 × 10^−34^ J s is Planck's constant, and *N*_A_ = 6.022 × 10^23^ is Avogadro's number.

*E*_a_ was determined from the slope of the Arrhenius plots (Fig. 8a and b), while Δ*H*_a_ and Δ*S*_a_ were determined from the slope and intercept of the transition state plots (Fig. 8c and d), respectively. The obtained values of these activation parameters for both inhibitors are summarized in Table 5.

**Table 5:** Thermodynamic parameters for the S235 steel samples after 24 h immersion in 1 M HCl solution with and without addition of the OCIC of APhBI and HPhBI

Solutions	*E*_a_ (kJ mol^−1^)	Δ*H*_a_ (kJ mol^−1^)	Δ*S*_a_ (J mol^−1^ K^−1^)
Blank	109.51	106.95	94.70
3 mM APhBI	146.44	143.88	202.28
3 mM HPhBI	145.36	142.81	200.03

Table 5 shows that the addition of the OCIC of both inhibitors resulted in increased energy barrier for the corrosion process (*i.e.* higher *E*_a_) compared with the uninhibited solution, leading to lower corrosion susceptibility of the S235 steel samples in 1 M HCl solution.^63^ The corrosion process was found to be endothermic, independently from the presence of each inhibitor. The increase of Δ*H*_a_ with the addition of the inhibitors is indicative of their ability to mitigate corrosion.^63^ The same trend was also observed for the variation of Δ*S*_a_ in the presence of the inhibitors owing to the replacement of water molecules with the inhibitors' molecules during the adsorption process.^64^

### 3.4 UV-Vis measurements

**Fig. 9:**
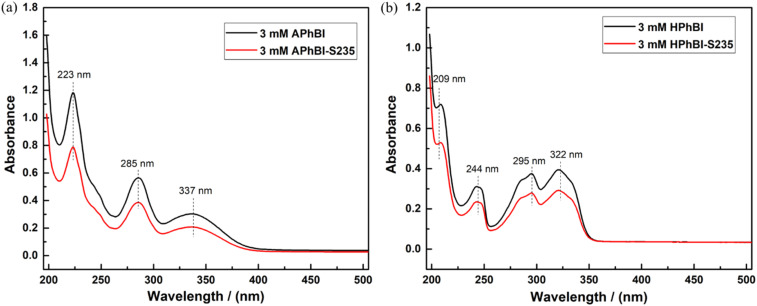
UV-Vis absorption spectra of 3 mM solutions of (a) APhBI and (b) HPhBI before and after 24 h immersion of S235 steel samples.

Fig. 9 presents the UV-Vis spectra of the 3 mM APhBI and HPhBI solutions before and after immersion of the S235 steel samples for 24 h at 298 K. For the solutions of both inhibitors the π–π* and n–π* transitions based on aromatic rings and lone pairs on heteroatoms were observed at 223, 285, and 337 nm for APhBI (Fig. 9a), while for HPhBI (Fig. 9b) at 209, 244, 295, and 322 nm.^65,66^

After the immersion of the S235 steel samples a decrease in absorption intensity, without significant peak shifts, occurred. This decrease in peak's intensity indicates a decrease of the inhibitors' concentration compared with the same solutions before immersion, due to the interaction of the molecules of these inhibitors with the surface of the S235 steel samples. The interaction can lead to the formation of a passivating surface layer on S235 steel through coordinative bonds, physisorption or chemisorption.^56,65^

### 3.5 Surface characterization of the S235 steel samples

#### 3.5.1 ATR-FTIR analysis

**Fig. 10:**
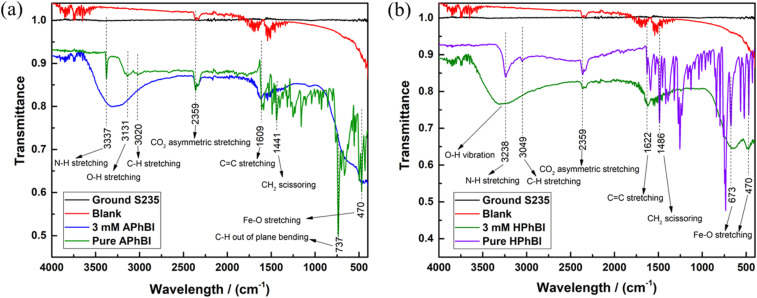
ATR-FTIR spectra of the S235 steel samples after 72 h immersion at 298 K in 1 M HCl solutions with and without addition of the optimum concentration of (a) APhBI and (b) HPhBI. The spectra of ground S235 and pure inhibitors are also presented.

The ATR-FTIR spectra of the S235 steel samples immersed for 72 h in 1 M HCl solutions with and without addition of the optimum concentration (*i.e.* 3 mM) of both inhibitors are presented in Fig. 10. For comparison, the spectra of the pure solid APhBI and HPhBI compounds, as well as of the ground S235 steel samples are also included.

The presence of aromatic double bonds is confirmed by the C=C stretching peak at 1609 cm^−1^ (ref. 67) for samples immersed in APhBI-containing solutions (Fig. 10a) and at 1622 cm^−1^ for samples in HPhBI-containing solutions (Fig. 10b). In addition, the N–H stretching peak at 3337 cm^−1^ (ref. 68) (Fig. 10a), confirmed the presence of the amino group for APhBI-containing solution, while the same vibrations are seen at 3238 cm^−1^ (Fig. 10b) when HPhBI was added. On the other hand, samples immersed in HPhBI-containing solutions (Fig. 10b) exhibited a broader peak at 3240 cm^−1^,^69^ a characteristic O–H stretching vibration of phenolic hydroxyl groups. Aromatic C–H vibration and CH_2_ scissoring are also present for both inhibitors. Finally, the above-mentioned peaks combined with the Fe–O stretching vibrations observed at 470 cm^−1^ (ref. 66) confirmed the adsorption of both inhibitors on the surface of the S235 steel samples.

#### 3.5.2 SEM analysis

**Fig. 11:**
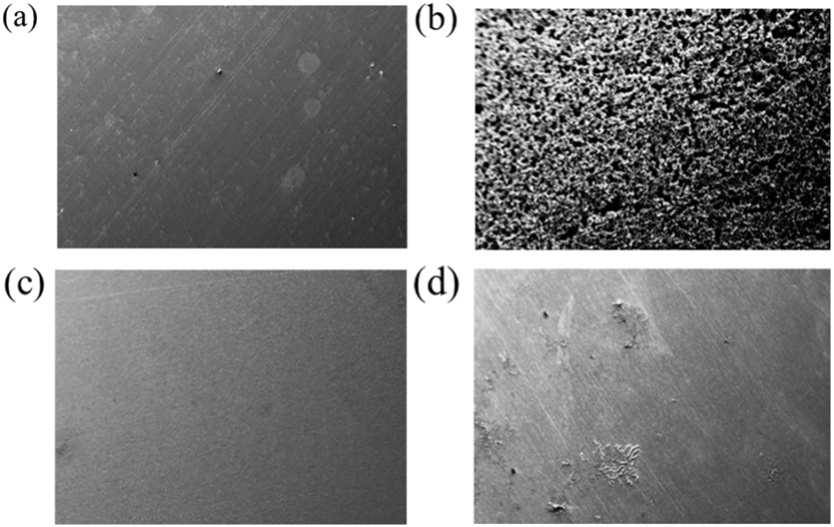
SEM micrographs of the ground S235 steel samples: (a) before immersion, (b–d) after immersion in 1 M HCl solution, without addition of the inhibitors and with addition of 3 mM APhBI, and HPhBI, respectively.

The impact of introducing 3 mM APhBI and HPhBI, on the surface morphology of the S235 steel samples following a 72 hour immersion in 1 M HCl solution at 298 K, was examined *via* scanning electron microscopy (SEM) analysis. Fig. 11 presents SEM images of the grounded S235 steel sample pre-immersion, as well as of the samples post-immersion in 1 M HCl solution with and without addition of 3 mM of each inhibitor. The images of the grounded S235 steel sample (Fig. 11a) displayed surface irregularities attributed to inherent material defects and grinding marks resulting from the grinding process. The SEM image of the sample immersed in uninhibited 1 M HCl solution (Fig. 11b) demonstrates marked surface degradation, revealing general corrosion. Conversely, the SEM images of the samples immersed in 1 M HCl solution with the addition of inhibitors (*i.e.* 3 mM) shown in Fig. 11c and d, exhibit a notably smoother surface, indicating decreased corrosion activity.

This observation is supported by electrochemical measurements and ATR-FTIR spectroscopic data, providing compelling evidence for the adsorption of APhBI and HPhBI molecules on the surface of the S235 steel samples.

### 3.6 Theoretical analysis

#### 3.6.1 DFT calculations

**Fig. 12:**
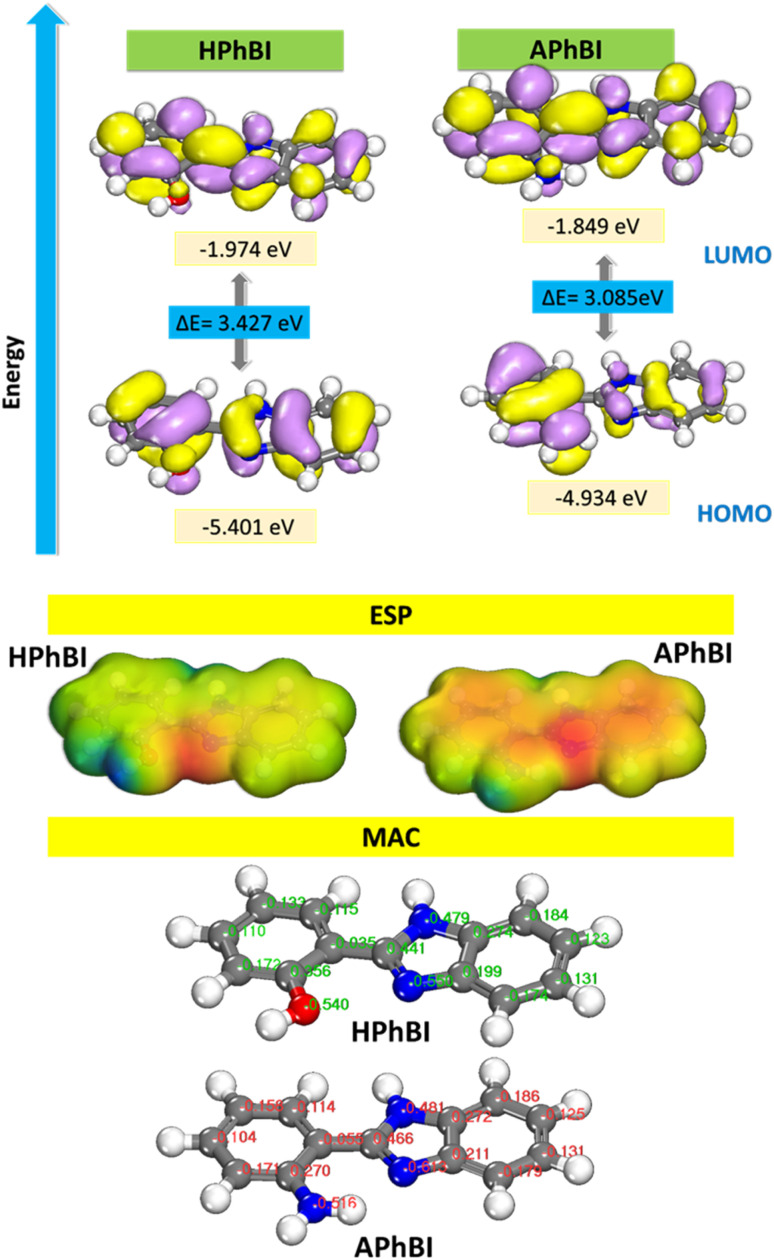
HOMO, LUMO, ESP and MAC of the studied inhibitors.

Fig. 12 presents the frontier molecular orbitals (FMOs), which play a crucial role in determining a molecule's stability.^70–72^ The highest occupied molecular orbital (HOMO) is the molecular orbital with the highest energy level that contains electrons.

Molecules with high HOMO energy tend to donate electrons, making them effective nucleophiles. Conversely, the lowest unoccupied molecular orbital (LUMO) is the molecular orbital with the lowest energy level and remains unoccupied by electrons.^73^ A low LUMO energy indicates that the molecule can readily accept electrons, classifying it as electrophilic.^44,74,75^

Fig. 12 illustrates that the frontier molecular orbitals (FMOs) are primarily localized on the π-electrons of the benzimidazole ring as well as on the molecule's heteroatoms (oxygen and nitrogen). Table 6 shows that a low HOMO energy (*E*_HOMO_) corresponds to a reduced tendency of the molecule to donate electrons. Additionally, a large energy gap (Δ*E*) between HOMO and LUMO suggests that the molecules have limited polarizability, indicating greater stability and lower reactivity. The electrostatic potential (MEP) map provides insight into the spatial distribution of electron density on the molecular surface, which is essential for identifying electrophilic and nucleophilic regions. In the MEP visualization red regions indicate areas of high electron density and negative potential (repulsion), blue regions signify partial positive charge and strong attraction, light blue areas suggest electron deficiency, yellow regions denote slight electron surplus, and green areas represent neutral charge distribution. The ESP map reveals a significant accumulation of negative charge around heteroatoms, highlighting their susceptibility to nucleophilic interactions.

**Table 6:** Quantum descriptors for the HPhBI and APhBI inhibitors

Parameter	HPhBI	APhBI
Electronegativity (*χ*)	3.6875	3.3915
Global hardness (*η*)	1.7135	1.5425
Chemical potential (*π*)	−3.6875	−3.3915
Global softness (*σ*)	0.5836	0.6483
Global electrophilicity (*ω*)	3.9678	3.7285
Electrodonating (*ω*−) power	6.0257	5.6170
Electroaccepting (*ω*+) power	2.3382	2.2255
Net electrophilicity (Δ*ω*±)	2.1723	2.0475
Fraction of transferred electrons (Δ*N*)	−0.1335	−0.0524
Energy from inhb to metals (Δ*N*)	0.0305	0.0042
Δ*E* back-donation	−0.4284	−0.3856

The Mulliken atomic charges (Fig. 12) provide valuable insight into the electron distribution within the APhBI and HPhBI molecules, which directly influences their behavior as corrosion inhibitors.^70,72,76–78^ The effectiveness of a molecule in inhibiting corrosion is closely linked to its ability to donate or accept electrons when interacting with a metal surface. The nitrogen atoms (N7, N9, N16) and oxygen (O16 in HPhBI) exhibit significantly negative Mulliken charges (−0.481 to −0.613). These electronegative atoms act as electron donors, facilitating strong adsorption onto the metal surface, thereby enhancing corrosion inhibition.^77,79^ In particular, N9 in APhBI (−0.613) is more negative than in HPhBI (−0.557), suggesting stronger electron donation capability in the former. APhBI has a slightly stronger ability to donate electrons due to the higher negative charge on N9. This suggests it may form more stable adsorption layers on the metal surface, leading to enhanced corrosion inhibition. HPhBI, on the other hand, has a more balanced charge distribution, particularly on C15, which may influence its solubility and adsorption characteristics. The electrostatic interactions between the inhibitor molecules and the metal surface are governed by these charge differences, influencing their overall corrosion protection effectiveness. To explore the relationship between molecular structure and the corrosion inhibition efficiency of these compounds, we employed Density Functional Theory (DFT) calculations to determine key quantum chemical parameters.^77,80,81^ The computed results are summarized in Table 6.

HPhBI (*χ* = 3.6875) has a higher electronegativity than APhBI (*χ* = 3.3915), indicating that it has a stronger tendency to attract electrons. The chemical potential (*π*) follows the same trend (*π*(HPhBI) = −3.6875 *vs. π*(APhBI) = −3.3915), meaning HPhBI is more inclined to accept electrons from the metal. This suggests that HPhBI may have slightly stronger physisorption interactions, while APhBI may exhibit stronger chemisorption due to lower electronegativity.^77,82^ Global hardness (*η*) is lower in APhBI (*η* = 1.5425) than in HPhBI (1.7135), indicating that APhBI is more reactive and can donate electrons more easily. Global softness (*σ*) is higher in APhBI (0.6483) than in HPhBI (0.5836), reinforcing its ability to interact more efficiently with the metal surface.^83^ Since corrosion inhibitors with low hardness and high softness tend to be more effective, APhBI appears to have a better inhibition potential. For the Δ*N* values below 3.6, the inhibition effectiveness enhances as the electron transport capacity to the metal surface augments.^37,84^ HPhBI has slightly higher electrophilicity (*ω* = 3.9678) than APhBI (*ω* = 3.7285), meaning HPhBI is more prone to accept electrons. However, electrodonating power (*ω*−) is higher in HPhBI (6.0257) than APhBI (5.6170), suggesting HPhBI can release electrons more efficiently, which enhances its interaction with the metal. The net electrophilicity difference (Δ*ω*±) is also slightly higher for HPhBI (2.1723 *vs.* 2.0475), which indicates that HPhBI has a slightly stronger electron exchange capability. The Δ*N* values are below 3.6 for both molecules, confirming effective electron transfer from the inhibitors to the metal surface, which promotes strong adsorption and protective layer formation.^39,85^ HPhBI (−0.1335) has a higher absolute Δ*N* compared to APhBI (−0.0524), suggesting that HPhBI donates more electrons to the metal surface.^49,50^ However, the energy transfer from inhibitor to metal (Δ*N*) is significantly lower in APhBI (0.0042) compared to HPhBI (0.0305), indicating that APhBI exhibits stronger adsorption stability. The Δ*E* back-donation values for both inhibitors are negative (Δ*E*(HPhBI) = −0.4284, Δ*E*(APhBI) = −0.3856), which suggests that both molecules can accept electrons back from the metal surface. A more negative value (−0.4284 for HPhBI) indicates better electron back-donation capability, enhancing stability. Overall, both HPhBI and APhBI can act as highly effective corrosion inhibitors. The HPhBI inhibitor exhibits a greater electron-donating ability (higher Δ*N*), slightly higher electronegativity, and an enhanced capacity to accept electrons, which favors physisorption interactions with the metal surface. In contrast, the APhBI inhibitor demonstrates lower global hardness, higher softness, and a lower Δ*N*, suggesting a stronger inclination for chemisorption.

#### 3.6.2 MC and MD simulations

The adsorption energy (*E*_ads_) can be determined using eqn (14) for the molecule under investigation. This equation serves as a fundamental tool for quantifying the strength and stability of the adsorption process between the inhibitor and the metal surface.^38,49^ A more negative *E*_ads_ value indicates stronger adsorption, suggesting a more effective corrosion inhibitor, as it implies greater binding affinity and stability of the protective layer on the metal surface.^86^ Understanding *E*_ads_ is crucial in evaluating the inhibitor's efficiency and its mechanism of interaction, whether through physisorption (weaker electrostatic interactions) or chemisorption within the system:^80,87^

14 *E*_adsorption_ = *E*_Fe(110)‖inhibitor_ − (*E*_Fe(110)_ + *E*_inhibitor_)

**Fig. 13:**
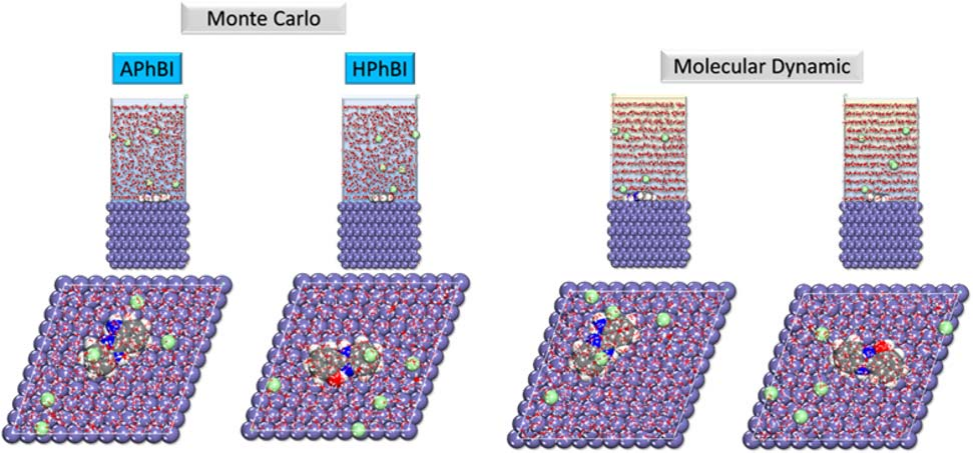
MC and MD simulations for the system configurations of the inhibitors.

Following the completion of the Monte Carlo (MC) simulation, depicted in Fig. 13, a thorough analysis was conducted to ensure the accuracy of the molecule's adsorption geometry. This evaluation was guided by previous studies, aligning the findings with established adsorption models to validate the simulation's reliability. To assess the stability of the MC simulation, the energy values at the simulation's conclusion were compared to those at the start, and the differences were carefully examined. Minimal fluctuations in energy indicate that the system has reached equilibrium, confirming the robustness and precision of the adsorption simulation. Fig. 13 offers a comprehensive visualization of the inhibitor molecules in their adsorbed state, providing clear insights into their structural arrangement on the metal surface.^48,84^ During the Molecular Dynamics (MD) simulations, it was observed that the inhibitor molecules self-assemble into a protective layer on the Fe(110) surface, aligning in a way that maximizes interactions with oxygen (O) and nitrogen (N) atoms.^38,49,50,84^ This strategic orientation enhances adsorption stability and corrosion resistance.

Furthermore, Fig. 13 illustrates the attachment of the studied compounds to the Fe(110) surface, emphasizing the role of heteroatoms in adsorption. The findings confirm that the inhibitor molecules effectively anchor themselves to the Fe(110) sites, reinforcing the formation of a stable protective barrier against corrosion.

**Fig. 14:**
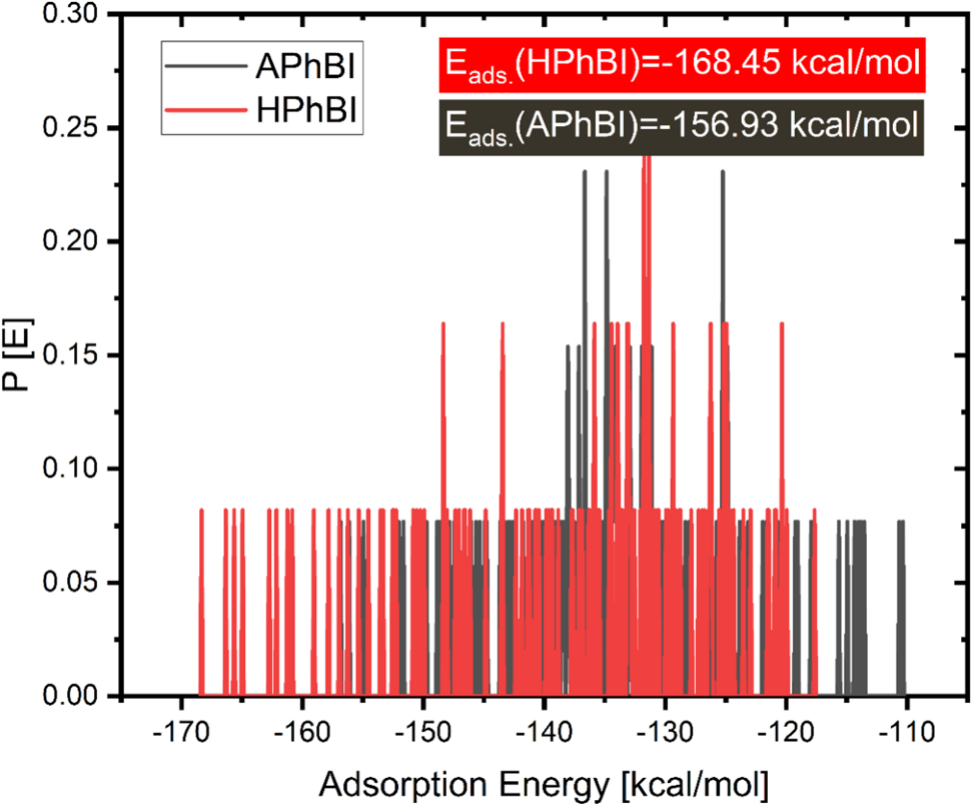
Distribution of the adsorption energies for the neutral and protonated form of the inhibitor as obtained *via* MC simulations.

**Fig. 15:**
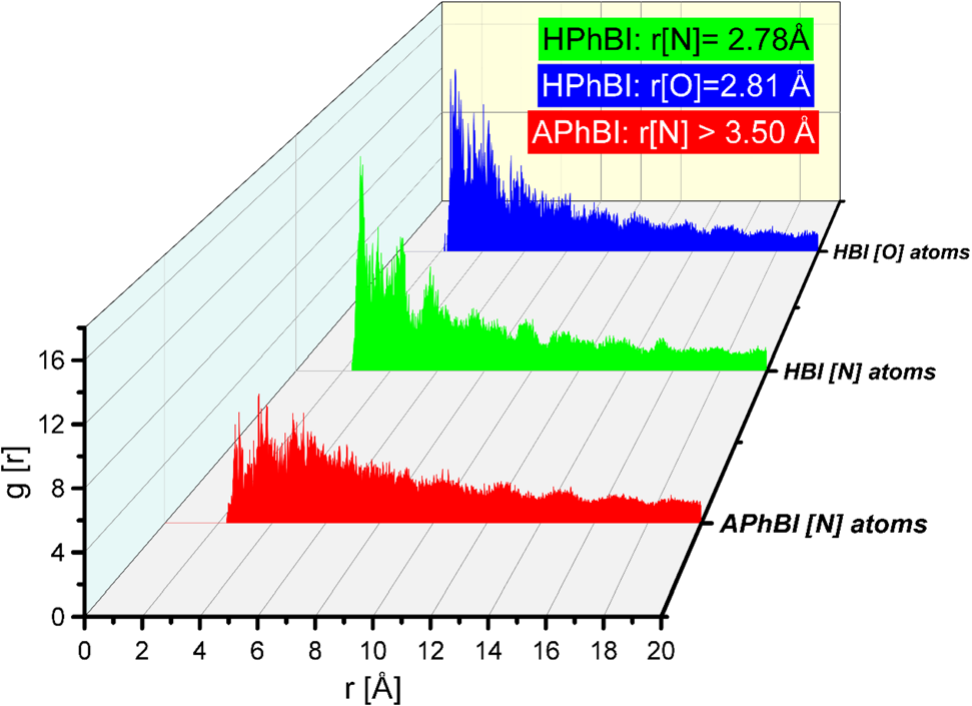
Distribution of the adsorption energies for the neutral and protonated form of the inhibitor as obtained *via* MC simulations.

The adsorption of inhibitor molecules onto the substrate surface results in a significant adsorption energy (*E*_ads_), as illustrated in Fig. 14. This high *E*_ads_ value indicates a strong interaction between the inhibitor and the metal surface, leading to the formation of a stable and durable protective layer.^37,49^ The robust adsorption ensures effective shielding of the metal from corrosion, reinforcing the importance of molecular interactions in corrosion inhibition. The metal substrate plays a pivotal role in this process, as the protective layer formation mitigates direct exposure to corrosive agents. The strength of the adsorption interaction determines the stability and longevity of the inhibitor's protective effect. Molecular Dynamics (MD) simulations are widely recognized for their precision in modelling adsorption dynamics. During the NVT ensemble simulation, which was conducted over several hundred picoseconds, the inhibitors exhibited a tendency to adopt a flattened configuration upon interacting with the Fe surface (as shown in Fig. 14).^50,84^ This flattening effect becomes increasingly pronounced with extended simulation times, further confirming the strong adhesion and stability of the inhibitor molecules on the Fe(110) surface. To further investigate the adsorption behaviour of corrosion inhibitors on metal surfaces, the Radial Distribution Function (RDF) method is employed. Fig. 15 presents the RDF analysis, which provides quantitative insights into the spatial distribution of inhibitor molecules above the metal's surface.^49,79^ This method is efficient and straightforward, offering valuable data on adsorption dynamics without requiring complex computational procedures.

The Radial Distribution Function (RDF) is a fundamental analytical tool in Molecular Dynamics (MD) simulations, widely used to examine the interactions between inhibitor molecules and metal surfaces.^76,88,89^ Extensive research underscores its importance in understanding adsorption behaviour, particularly in distinguishing chemisorption and physisorption mechanisms. Studies indicate that for chemisorption, the RDF peak generally appears within the 1 to 3.5 Å range, signifying strong molecular attachment *via* covalent or coordination interactions.^72,74,79,90^ In contrast, physisorption typically exhibits an RDF peak at distances beyond 3.5 Å, indicative of weaker electrostatic interactions. As illustrated in Fig. 5, the RDF analysis for the inhibitor–metal interaction reveals that the interaction distance between Fe and the inhibitor remains within 3.5 Å, suggesting strong surface adsorption and a high binding affinity.^44,71,77,91^ Furthermore, the RDF peak position indicates that the inhibitor predominantly interacts with Fe through its oxygen (O) and nitrogen (N) atoms, reinforcing the role of these heteroatoms in enhancing adsorption strength and corrosion inhibition efficiency.

In conclusion, the theoretical predictions obtained from DFT, MC, and MD simulations complement and reinforce the experimental findings. The high adsorption energy values and Mulliken charge distributions suggest strong electron-donating behaviour of the inhibitors, aligning with the observed high inhibition efficiencies in electrochemical measurements. The flattening of inhibitor molecules over the Fe(110) surface in MD simulations mirrors the dense protective films observed in SEM images. Furthermore, the trend in inhibition efficiency across temperatures correlates with Δ*N* values and global softness parameters, providing a consistent mechanistic rationale for the adsorption mode (physisorption *vs.* chemisorption). This synergy between theory and experiment highlights the robustness of the inhibitors and supports the structure–activity relationships established.

## 4. Conclusions

A combined experimental and theoretical approach was employed in this study to investigate the corrosion susceptibility of the S235 steel samples immersed in 1 M HCl, at 298–318 K, with additions of 2-(2-aminophenyl)-1*H*-benzimidazole (APhBI) and 2-(2-hydroxophenyl)-1*H*-benzimidazole (HPhBI). The following conclusions can be drawn:

• The weight loss and electrochemical measurements confirmed that the addition of both APhBI and HPhBI increased the corrosion resistance of the S235 steel samples. No significant difference in corrosion inhibition performance of both derivatives was observed. The highest inhibition efficiencies, *i.e.* 87.1 and 85.1%, were achieved upon addition of 3 mM of APhBI and HPhBI, respectively.

• The corrosion inhibition efficiencies of both derivatives increased with increasing their concentration but decreased with increasing immersion time and with increasing temperature in the 298–318 K temperature range.

• The addition of potassium iodide, formic acid, paraformaldehyde and propargyl alcohol in the inhibitor to intensifier ratio of 3 : 1, further improved the corrosion inhibition performance of APhBI. Meanwhile, for HPhBI the same effect was achieved only upon addition of potassium iodide and propargyl alcohol. The latter was found to be the most effective intensifier for both inhibitors.

• Electrochemical measurements indicated that both inhibitors predominantly affect the cathodic corrosion reaction. Moreover, the corrosion process of the S235 steel samples in inhibited 1 M HCl solution is kinetically controlled.

• Attenuated total reflectance Fourier-transform infrared spectroscopy, ultraviolet-visible spectroscopy, and scanning electron microscope measurements confirmed the adsorption of both derivatives on the steel surface. Thermodynamic studies showed that the adsorption process was a combination of physisorption and chemisorption and obeyed the Langmuir isotherm.

• The Mulliken charge analysis shows that the heteroatoms (N and O) act as active adsorption sites, promoting strong molecular interactions with the Fe(110) surface. Additionally, the electrostatic potential (ESP) and frontier molecular orbitals (FMOs) provide insight into the electronic properties influencing adsorption behaviour.

• The MC and MD simulations confirm the formation of a protective inhibitor layer on the metal surface, with the molecules adopting a favourable adsorption geometry that maximizes interactions with Fe atoms. The adsorption energy calculations demonstrate that both inhibitors exhibit strong adsorption affinities.

## Author contributions

Conceptualization, K. X.; methodology, K. X., M. F., J. C., K. X., E. K., and A. L.; software, M. F. and A. B.; validation, K. X.; formal analysis, K. X., M. F., B. S., K. X., J. C., E. K., and A. L.; investigation, M. F. K. X.; resources, K. X., K. X., A. B., J. C.; data curation, M. F. and K. X.; writing—original draft preparation, M. F., K. X., A. B., B. S., K. X., J. C., E. K., and A. L.; writing—review and editing, K. X., M. F., A. B., B. S., K. X., J. C., E. K., and A. L.; visualization, M. F.; supervision, K. X.; project administration, K. X.; funding acquisition, K. X. All authors have read and agreed to the published version of the manuscript.

## Conflicts of interest

The authors declare no conflict of interest. The funders had no role in the design of the study; in the collection, analyses, or interpretation of data; in the writing of the manuscript; or in the decision to publish the results.

## Data Availability

The data presented in this study are available on reasonable request from the corresponding author.
